# Two short sequences in *OsNAR2.1* promoter are necessary for fully activating the nitrate induced gene expression in rice roots

**DOI:** 10.1038/srep11950

**Published:** 2015-07-07

**Authors:** Xiaoqin Liu, Huimin Feng, Daimin Huang, Miaoquan Song, Xiaorong Fan, Guohua Xu

**Affiliations:** 1 State Key Laboratory of Crop Genetics and Germplasm Enhancement; 2 MOA Key Laboratory of Plant Nutrition and Fertilization in Lower-Middle Reaches of the Yangtze River, Nanjing Agricultural University, Nanjing 210095, China

## Abstract

Nitrate is an essential nitrogen source and serves as a signal to control growth and gene expression in plants. In rice, OsNAR2.1 is an essential partner of multiple OsNRT2 nitrate transporters for nitrate uptake over low and high concentration range. Previously, we have reported that −311 bp upstream fragment from the translational start site in the promoter of *OsNAR2.1* gene is the nitrate responsive region. To identify the *cis*-acting DNA elements necessary for nitrate induced gene expression, we detected the expression of beta-glucuronidase (GUS) reporter in the transgenic rice driven by the *OsNAR2.1* promoter with different lengths and site mutations of the 311 bp region. We found that −129 to −1 bp region is necessary for the nitrate-induced full activation of *OsNAR2.1*. Besides, the site mutations showed that the 20 bp fragment between −191 and −172 bp contains an enhancer binding site necessary to fully drive the *OsNAR2.1* expression. Part of the 20 bp fragment is commonly presented in the sequences of different promoters of both the nitrate induced *NAR2* genes and nitrite reductase *NIR1* genes from various higher plants. These findings thus reveal the presence of conserved *cis*-acting element for mediating nitrate responses in plants.

Nitrate in plants serves as a nutrient as well as a signal which induces changes in growth and gene expression^1^,^2^,^3^,^4^,^5^,^6^,^7^. Large number of the genes in plants are involved in nitrate responses and regulation. When plants were exposed to nitrate, expression of nitrate transporters genes (*NRT/NAR*) and nitrate assimilation related genes *(NIA*, *NiR*) were immediately induced or enhanced^8^,^9^,^10^,^11^,^12^,^13^,^14^. Genome-wide gene expression analyses have showed that expression of a wide spectrum of the genes involved in nutrient uptake, metabolism, growth and development are rapidly altered by nitrate^15^,^16^,^17^,^18^,^19^,^20^,^21^.

The *cis*-regulatory modules in responses to nitrate supply are emerging in plants. To date, several putative transcription factors linking to nitrate regulation have been reported, including NPL7^22^,^23^, SPL9^24^, TGA1 and TGA4^25^. In addition, a handful of *cis*-acting element(s) in the promoters of nitrate regulated genes has been identified. In *Arabidopsis*, a 150 bp fragment from the promoter of a nitrate transporter gene *AtNRT2.1* was shown to be a nitrate specific regulation region^26^. A 43 bp sequence containing the fragment 5′-GACcCTTN_10_AAG-3′ in the promoter of a nitrite reductase gene *AtNIR1* has emerged as nitrate-responsive *cis*-regulatory elements^27^. A 180 bp fragment from the promoter of nitrate reductase gene *AtNIA1* contains three elements corresponding to the predicted binding motifs of nitrate enhancer^28^. However, motif analysis showed that these reported fragments and the putative nitrate responsive *cis*-acting elements [5′-GATA-3′^29^,^30^, 5′-A(C/G)TCA-3′^31^, 5′-GACtCTTN_10_AAG-3′^27^] are not commonly presented in the promoters of nitrate responsive genes from different plant species^32^. There might be multiple *cis*-elements involved in different response of the genes to nitrate and nitrogen (N) supplies in plants^27^.

During the past two decades, the nitrate transport and signaling in plants have been well characterized^24^,^33^,^34^,^35^. A nitrate inducible gene, *NAR2.1* (nitrate assimilation related gene), has been defined as the gene encoding nitrate accessory protein^32^,^36^,^37^. *NAR2* is mainly expressed in roots and induced by nitrate and suppressed by ammonium^32^,^38^,^39^. In Arabidopsis, expression of *NAR2.1* is required for the activities of multiple NRT2 nitrate transporters for both constitutive and inducible high affinity nitrate uptake^40^,^41^,^42^,^43^. The expression of *AtNAR2.1* and *AtNRT2.1*, *AtNRT2.2* was coordinately inducted by low external nitrate concentration and sudden N deprivation, and suppressed by high nitrate supply^40^,^41^. In rice, OsNAR2.1 is a partner protein interacting with OsNRT2.1, OsNRT2.2, and OsNRT2.3a, affecting nitrate uptake over low and high concentration ranges^32^,^44^,^45^. The expression of *OsNAR2.1*, *OsNRT2.1*, *OsNRT2.2* and *OsNRT2.3a* genes was coordinately inducted by both low and high nitrate concentrations in roots, and knockdown of *OsNAR2.1* in turn synchronously suppressed expression of *OsNRT2.1*, *OsNRT2.2* and *OsNRT2.3a*^44^.

The *cis*-acting regulatory components for sensing nitrate in rice were scarcely reported. Previously, we have shown that a region from the position –311 to –1 bp, relative to the translation start site in the promoter of *OsNAR2.1*, was found to contain the nitrate responsive *cis*-element(s), while no similar *cis*-element(s) is presented in the promoters of *OsNAR2.1* and *OsNRT2s*^32^. In this study, we revealed that the 20 bp sequence between –191 and –172 bp in addition to –129–1 bp region contains the nitrate enhancer element which is required to drive *OsNAR2.1* expression. Our results demonstrated that both a minimal fragment and conserved *cis*-acting element are necessary for mediating the nitrate induced gene expression in rice.

## Results

### A 192 bp region at the upstream from translational start codon of *OsNAR2.1* gene is sufficient for fully mediating the nitrate induced expression

**Figure 1:**
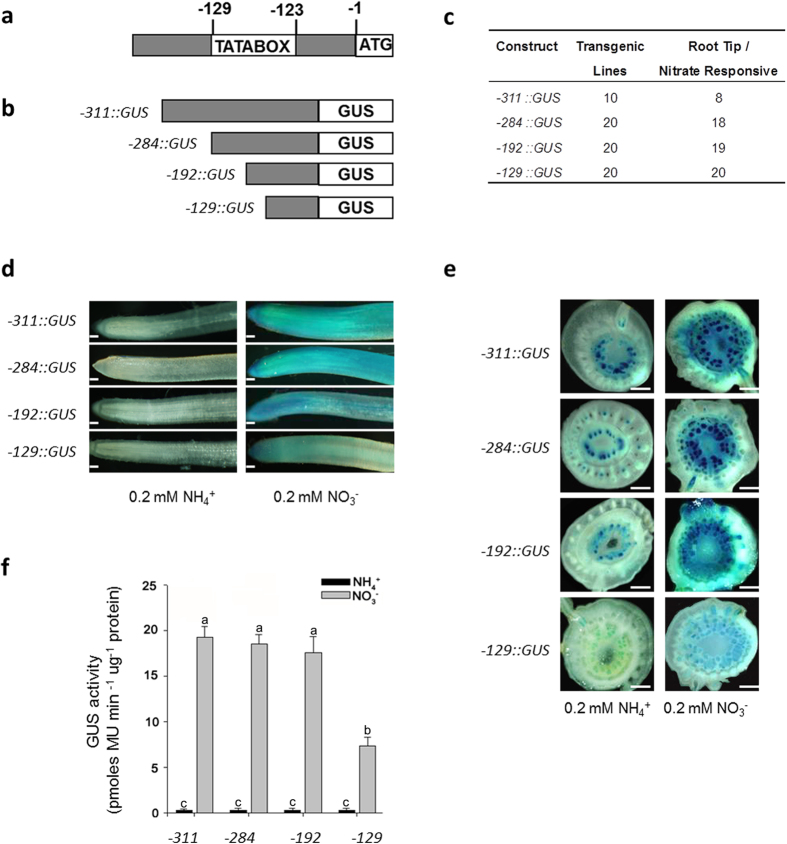
Deletion analysis of the *OsNAR2.1* 311 bp promoter fragment in rice transgenic lines for nitrate enhancer elements. (**a**) TATABOX position in *OsNAR2.1* promoter. (**b**) Schematic representation of the diagram of binary cassettes fused the *OsNAR2.1* promoter fragments with GUS reporter gene. –311::GUS, –284::GUS, –192::GUS, and –129::GUS, represent the binary cassette with GUS under the control of the flanking region upstream of the translation start codon (ATG) of 311 bp, 284 bp, 192 bp, and 129 bp, respectively. (**c**) Analysis was performed on 10 and 20 independent lines transformed with each construct. (**d**,**e**) Histochemical analysis of GUS activity in the roots (**d**) and root-shoot junction (**e**) of the representative transgenic line grown in the nutrient solution containing 0.2 mM NH_4_^+^ or 0.2 mM NO_3_^−^ for seven days. Bars: 1 mm (**d**) and 0.5 mm (**e**). (**f**) Quantification of the root GUS activity. Analysis was performed on six independent transgenic lines grown in either ammonium or nitrate solution. Each GUS activity assay was performed for each line as described in “Materials and Methods”. a, b and c indicate the significant difference at p < 0.05 between the four lengths of *OsNAR2.1* promoter treated with different forms of N. Values are mean ± SE of six biological replicates.

Previously, we identified that the –311/–1 bp region of *OsNAR2.1* promoter contains the nitrate regulated element(s)^32^. To further dissect the nitrate response *cis*-acting element(s), we first made different deletions from the upstream of TATA-box region (–129/–123 bp) and generated three fragments of –284/–1 bp, –192/–1 bp and –129/–1 bp of *OsNAR2.1* promoter (Fig. 1a,b). These truncated promoter regions were respectively fused with beta-glucuronidase (GUS) reporter gene and transformed into rice (cv. Nipponbare). We generated twenty independent transgenic lines for each of the constructs harboring the different lengths of *OsNAR2.1* promoter (Fig. S1), and nearly all of these lines had the responses of the GUS reporter to nitrate in their roots (Fig. 1c). The histochemical staining of GUS reporter in the transgenic lines showed that these promoters were not activated by exogenously supplied ammonium (Fig. 1d–f). In contrast, the nitrate induced GUS expression controlled by all these promoters and the expression pattern in both the root and root-shoot junction were similar for the lines transformed with –311p::GUS, –284p::GUS, and –192p::GUS (Fig. 1d,e). However, the 129p::GUS transgenic lines showed a remarkably suppression of GUS activity compared with other transgenic lines (Fig. 1d,e). Quantitative analysis of GUS reporter enzyme activity in the transgenic rice roots confirmed the visible difference (Fig. 1f). Furthermore, qRT-PCR analysis using the roots of WT and the transgenic lines revealed that supply of nitrate in comparison to ammonium strongly elevated the levels of endogenous *OsNAR2.1* mRNAs (Fig. S2). No significant difference of the abundance of *OsNAR2.1* transcripts was observed between WT and the transgenic lines (Fig. S2), indicating that transforming the GUS report construct into rice did not affect the expression of endogenous *OsNAR2.1* gene. Although the –129/–1 bp promoter drove the GUS activity only about 40% of that by –192/–1 bp promoter, their expression patterns in both roots and root-shoot junction were similar to that obtained with the –311/–1 bp promoter (Fig. 1d,e; Fig. S1). The GUS expression patterns indicate that the *cis*-regulatory elements involved in nitrate induced gene expression locate in the –192/–1 bp region of the promoter, and the –192/–129 bp region might contain the nitrate *cis*-element(s) which are required for enhancing *OsNAR2.1* expression.

### A 129 bp region at the upstream from translational start codon of *OsNAR2.1* gene is essential for mediating basic nitrate response

**Figure 2:**
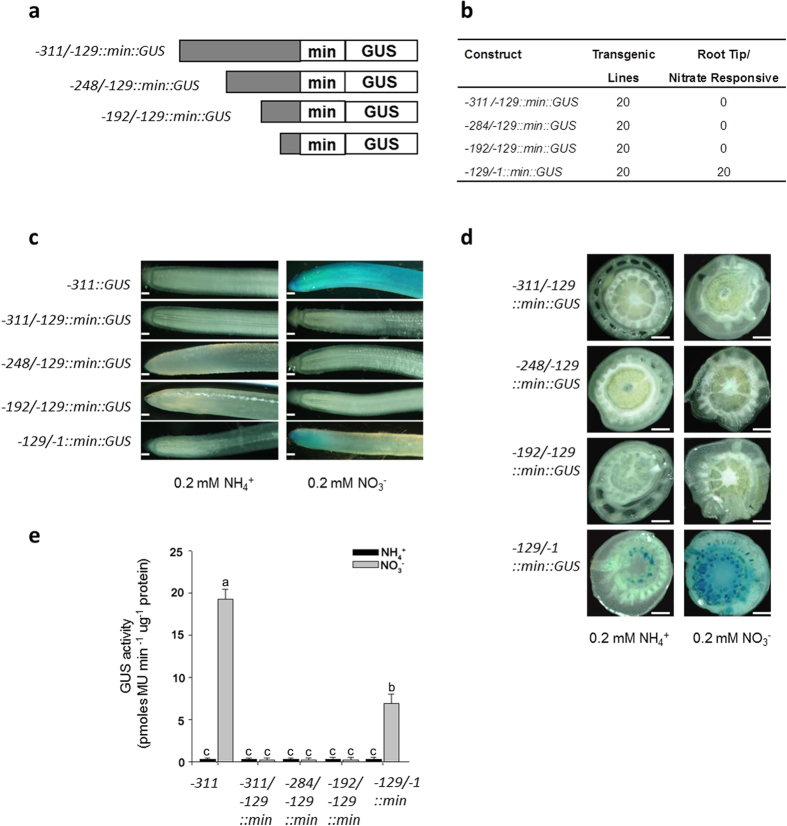
Identification of the region required for the nitrate response using the CaMV 35S minimal promoter. (**a**) Schematic representation of *cis*-activation of the CaMV 35S minimal promoter by sequences located upstream of the TATA box of *OsNAR2.1* in the transgenic lines. Constructs of –311/–129::min::GUS, –284/–129::min::GUS, –192/–129::min::GUS and –129/–1::min::GUS are the different promoter fragments fused with 35S minimal promoter and GUS reporter gene. (**b**) Analysis was performed on 20 independent transgenic lines for each construct. (**c**,**d**) GUS expression pattern by histochemical staining in the roots (c, Bars: 1 mm) and root-shoot junction (d, Bars: 0.5 mm). (**e**) Quantification of the root GUS activity. Analysis was performed on six independent transgenic lines grown in either ammonium or nitrate solution. Each GUS activity assay was performed for each line as described in “Materials and Methods”. a, b and c indicate the significant difference at p < 0.05 between the four lengths of *OsNAR2.1* promoter treated with different forms of N. Values are mean ± SE of six biological replicates.

To further characterize if the –129/–1 bp region is critical for *OsNAR2.1* to sensing nitrate supply, we generated three –129 bp deleted promoters with different lengths (–311/–129 bp, –284/–129 bp, –192/–129 bp) and the –129 bp promoter in which both CaMV 35S minimal promoter (min) and GUS reporter gene were fused in the constructs (Fig. 2a). Twenty independent transgenic lines for harboring each of the constructs were tested for detecting the GUS expression under either nitrate or ammonium supply condition (Fig. S1). It showed that all the three –129 bp deleted promoters of *OsNAR2.1* gene spanning –311/–129 bp, –284/–129 bp and –192/–129 bp region, respectively, lost the function in driving the nitrate induced GUS activity in both the roots and root-shoot junction (Fig. 2b–d). The quantitative GUS activity measurement confirmed the visible results (Fig. 2e). The insertion of the GUS construct with the different promoters did not affect the response of endogenous *OsNAR2.1* expression to nitrate (Fig. S2). In contrast, the transgenic lines expressing –129/–1::min::GUS containing the TATA-box (–129/–123 bp) showed nitrate induced GUS activity, even though the activity was much less stronger than that driven by the –311::GUS (Fig. 2c–e). These results suggest the sequence of –129/–1 bp indeed be required for the nitrate regulated expression of *OsNAR2.1*.

### The 20 bp sequence between −191 bp and −172 bp of the *OsNAR2.1* promoter contains the transcriptional enhancer element(s) for sensing nitrate supply

**Figure 3:**
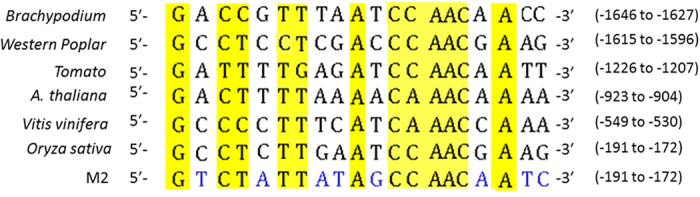
Analysis of the 20 bp conserved sequence in the promoters of *NAR2s* from different plant species. Alignment of conserved sequences in the *NAR2* gene promoters from *Brachypodium distachyon*, *Populus trichocarpa* (Western Poplar), *Solanum lycopersicum* (Tomato), *Arabidopsis thaliana*, *Vitis vinifera* (Grape), *Oryza sativa* (rice). M2, mutation of non-fully conserved 8 nucleotides in the sequence between –191 bp and –172 bp of *OsNAR2.1* promoter. The highly conserved nucleotides are highlighted with yellow color.

Since the nitrate induced GUS activity driven by the –129/–1 bp promoter was much less strong than that by the –192/–1 bp promoter of *OsNAR2.1* gene (Figs 1 and 2), the –192/–129 bp region may have the nitrate enhancer binding site for activating transcriptional expression. Interestingly, we found the 20 bp fragment of 5′-GCCTCTT(GAATCCAACG)AAG-3′ at the region between –191 bp and –172 bp of the *OsNAR2.1* promoter showed a high similarity with the motif 5′-GACTCTTN_10_AAG-3′ in the *AtNIR1* promoter which is critical for nitrate inducibility^27^. In addition, we found that the 20 bp sequence in *OsNAR2.1* promoter is relatively conserved in the putative promoters of *NAR2* genes from different plant species including Arabidopsis, bean, birch and tobacco (Fig. 3).

**Figure 4:**
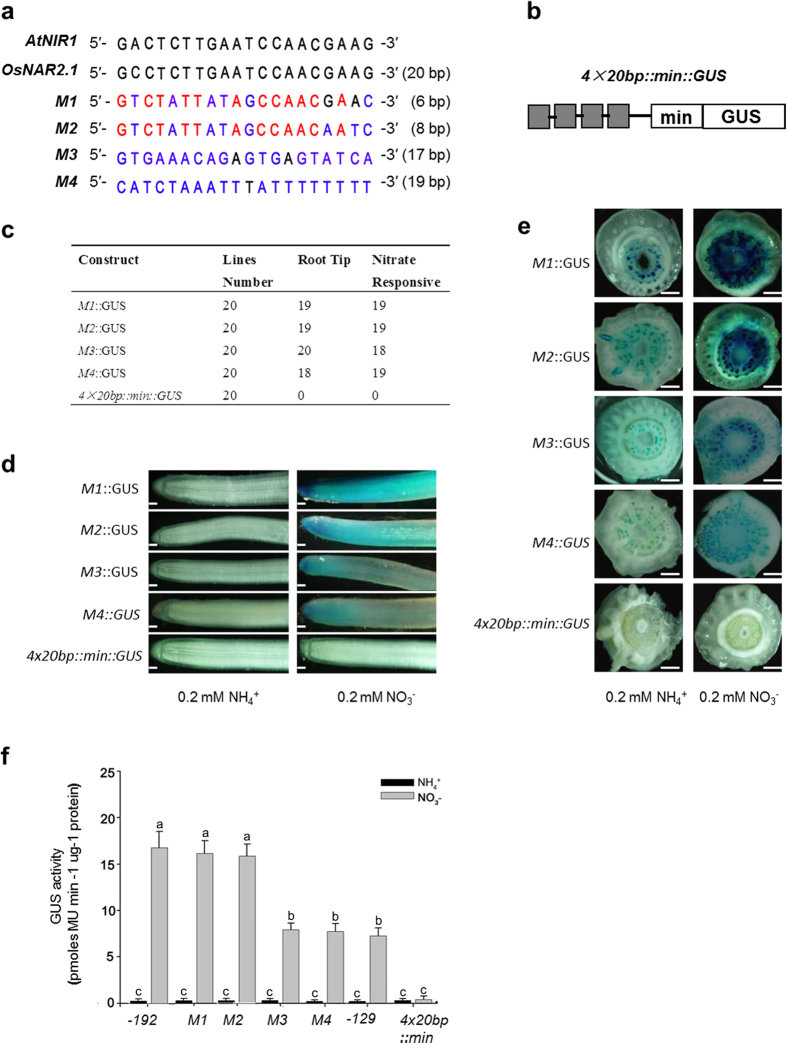
Analysis of the –191 bp to –172 bp sequence as a conserved transcription enhancer element in *OsNAR2.1* promoter. (**a**) Comparison of the essential nitrate enhancer element in *AtNIR1* gene and the 20 bp sequence with different mutations between –191 bp and –172 bp of *OsNAR2.1* promoter. M1, 6 bp mutation; M2, 8 bp mutation; M3, 17 bp mutation; M4, 19 bp mutation. The highly conserved nucleotides and mutated nucleotides are labeled with red and blue color, respectively. (**b**) The diagram of binary cassettes of the 4 × 20 bp::min::GUS, representation of a reporter construct with a synthetic promoter in which four copies of the 20-bp sequence are placed upstream of the 35S minimal promoter. (**c**) Analysis was performed on 20 independent transgenic lines for each construct. (**d**,**e**) GUS expression pattern by histochemical staining in the roots (c, Bars: 1 mm) and root-shoot junction (d, Bars: 0.5 mm) of M1::GUS, M2:GUS, M3::GUS, M4::GUS and 4 × 20 bp::min::GUS transgenic lines. (**e**) Quantification of the root GUS activity. Analysis was performed on six independent transgenic lines grown in either ammonium or nitrate solution. Each GUS activity assay was performed for each line as described in “Materials and Methods”. a, b and c indicate the significant difference at p < 0.05 between the site mutations and 4 × 20 bp::min::GUS of *OsNAR2.1* promoter treated with different forms of N. Values are mean ± SE of six biological replicates.

To test if this 20 bp-sequence functions as the putative nitrate enhancer element in the –192/–129 bp region, the effects of mutating this fragment on the promoter activity were examined. We generated four –192/–1 bp promoters with the 20 bp-sequence mutations of M1 (6 bp), M2 (8 bp), M3 (17 bp), M4 (19 bp) and one synthetic promoter with the fusion of four copies of the 20 bp sequence (4 × 20 bp) to the 35S minimal promoter (Fig. 4a,b). These point or site mutated or synthetic promoters were further fused with GUS reporter and transformed into rice. The GUS expression patterns of the transgenic lines are shown in Figure S1. Both histochemical staining and GUS activity measurement showed that the mutations in comparison to the native promoter did not change the very faint basal activity of the promoter under ammonium supply condition (Fig. 4c–f). Randomly point mutation of total 6 bp (M1) and 8 bps (M2) which are not completely conserved in the 20 bp-fragment of *NAR2.1* promoters from different species (Fig. 3) did not significantly alter the promoter activity in response to nitrate to drive GUS reporter (Fig. 4d–f). However, mutating most of the base pairs of the 20 bp fragment (M3 and M4) drastically decreased the promoter activity to drive GUS reporter under the same nitrate treatment (Fig. 4d–f). Interestingly, the M3 and M4 mutations resulted in the same activity of –192/–1 bp and native –129/–1 bp promoter of *OsNAR2.1* in responses to nitrate (Fig. 4d–f), indicating that the 20 bp sequence contains essential *cis*-element for enhancing the nitrate response of *OsNAR2.1* in rice. However, 4 × 20 bp::mini::GUS transgenic lines had no GUS activities under the same nitrate treatment (Fig. 4d–f), which implied that the 20 bp sequence itself is not enough for conferring the nitrate signal to induce the transcriptional expression.

## Discussion

Nitrate supply can trigger the rapid change of expression of the genes involved in nitrate uptake and assimilation, as well as their associated carbon and energy metabolism^20^,^28^,^46^. Although there are common responses of the genes encoding two components of high affinity nitrate transporters (NAR2.1 and its associated NRT2s) as well as a number of the genes encoding nitrate and nitrite reductase to different N forms, the identity of the nitrate regulatory factor(s) and conserved *cis*-acting element(s) were uncertain in plants^36^. In our current study, we analyzed the *OsNAR2.1* promoter and identified that both –129/–1 bp and 20 bp (–191/–172 bp) fragments upstream from the translational start site are necessary to mediate the nitrate induced gene activation in rice. The 20 bp sequence is relatively conserved among *NAR2* and *NIR1* genes from various plant genomes (Fig. 3).

*OsNAR2.1* in rice might be a key gene not only for nitrate uptake but also for the early sensing nitrate supply and transferring this signal to other nitrate responsive genes^32^,^37^. In this study, we detected that a short fragment of –192/–1 bp in the *OsNAR2.1* promoter contains the necessary *cis*-elements for responding to nitrate supply, while the –311/–193 bp fragment did not contribute to sense nitrate signal in mediating the gene expression (Fig. 1). Konishi and Yanagisawa (2010) compared the sequences of several nitrite reductase gene promoters from various higher plants and identified a conserved sequence motif 5′-GCCcCTTN_10_AAG-3′ as the putative nitrate responsive element (NRE)^27^. We found that the 20 bp sequence at –191/–172 bp region of the *OsNAR2.1* promoter, 5′-GCCTCTT(GAATCCAACG)AAG-3′, showed a high similarity with the NRE. However, this fragment is not presented in any of the putative promoters of nitrate responsive *OsNRT2* genes^32^. Since *OsNAR2.1* interacts with multiple *OsNRT2* members in both transcriptional and translational levels^44^,^45^, lack of the conserved motif in the promoters of *OsNRT2* genes indicates that either *OsNAR2.1* and these *OsNRT2* are regulated by different transcription factors or *OsNAR2.1* transfers the nitrate signal for upregulating the expression of *OsNRT2* members.

Mutations of the conserved sequence in the 20 bp motif of *NAR2* promoters (M3 and M4) from different plant species (Fig. 3) resulted in markedly suppression of the nitrate-responsive activity (Fig. 4), indicating that this motif might be a key sequence for the transcriptional enhancing and the sensing the nitrate supply. Konishi and Yanagisawa (2010) have defined that the 43 bp sequence containing the 20 bp conserved sequence is necessary for the full activation of the native *NIR1* promoter by nitrate. However in this study, its four copies fused to 35S minimum promoter did not have the function in mediating the nitrate induced gene expression (Fig. 4). In general, promoter activity is determined by the combined effects of many *cis*-elements^27^,^28^. Some of these binding sites mediate particular intercellular or intracellular stimuli, or developmental signals, and others function simply as an enhancer that is independent of a particular signaling pathway^27^,^28^. In this way, the 20 bp conserved sequence of *OsNAR2.1* promoter functions simply as an enhancer which is necessary for nitrate-responsive transcription (Fig. 4), while the 20 bp fragment itself is not enough for conferring the nitrate signal to induce the transcriptional expression. Interestingly, point mutations in not completely conserved positions (M1 and M2) did not affect the promoter activity in driving GUS expression (Fig. 4d–f). The expression pattern implies that the discontinuous 12 bp region in the motif (Figs 3 and 4) maybe the key biding site of transcription enhancer for mediating the nitrate regulation. Since the 12 bp are highly conserved among the promoters of known plant *NAR2* members (Fig. 3), it will be interesting to explore whether they perform a similar function for other *NAR2s* genes in different plant species.

Within the 20 bp of *OsNAR2.1* promoter (Fig. 3), the highly conserved sequence 5′-AATCCAAC-3′ has been reported to be specifically binding site with a GBF factor isolated from nuclear extracts of tomato and Arabidopsis^46^. In addition, the sequence 5′-CTCTT-3′ in the 20 bp region is putative nodulin consensus sequences as a *cis*-acting elements controlling expression of the root nodule-specific soybean leghemoglobin gene^47^,^48^. To date, no trans-acting factor that directly regulates the nitrate-responsive transcription have been identified in rice. Our identification of the relative conserved sequence will facilitate a search for a novel-type of transcription factor in sensing nitrate signaling.

The –129/–1 bp fragment of *OsNAR2.1* promoter could drive the nitrate induced gene expression (Fig. 1), while the fragment between –311/–129 bp was not able to *cis*-activate the transcription (Fig. 2), implicating that the 129 bp region also have the *cis*-regulatory elements in controlling the promoter activity. The GATA transcription factors have been predicted to be involved in regulating nitrate acquisition pathways^49^,^50^,^51^, while R2R3-MYB is involved in nitrate signaling^52^. Interestingly, the 129 bp *cis*-acting sequence contains the motif potentially being able to interact with GATA transcription factors and a binding site of the transcription factor R2R3-MYB (Table S1).

Some transcription factors bind multiple recognition sequences^53^,^54^,^55^. Multiple transcription factors function as a hub to perceive phosphate and mycorrhiza signals in plants have been well characterized^56^,^57^. For example, two conserved *cis*-acting elements, MYCS and P1BS, are involved in the regulation of mycorrhiza-activated phosphate transporters in eudicot species^57^. A single pair of the core motif in a large number of nitrate responsive genes is neither specific to nitrate responsive genes, nor common to all nitrate responsive genes and is randomly distributed throughout the genomes in both Arabidopsis and rice^52^. So, we deduced that the relative conserved 20 bp sequence in –192/–129 bp region is required to allow the enhancement of the GUS expression via the –129 bp fragment as combining sites of nitrate signal factor(s). However, whether these motifs are sufficient to confer the transcriptional regulation or need to interact with other elements remain unknown.

## Materials and Methods

### Construction of reporter vectors

The –311::GUS, –284::GUS, –192::GUS, and –129::GUS were obtained by fusing 311 bp, 284 bp, 192 bp, 129 bp fragments corresponding to the sequence located upstream of the initiating codon of *OsNAR2.1* to the b-glucuronidase (GUS) coding sequence using the primers showing in Table S2. The obtained DNA fragment for the *OsNAR2.1* promoter was digested with NcoI and HindIII. These cloned fragments were used to replace the 35S-promoter which was inserted at upstream of the 5′ end of the GUS reporter gene in the pCB302-35S-GUS vector^27^.

The 35S minimal promoter (min) is a 62 bp fragment with HindIII and Xho I sites located respectively at the 5′ and 3′ ends. Four copies of 20 bp (4 × 20 bp) were commercially synthesized by GenScript company. Chimaeric promoter constructs (–311/–129::min, –284/–129::min, –192/–129::min, –129/–1::min, 4 × 20 bp::min) were obtained by inserting PCR-amplified fragments corresponding to the –311/–129, –284/–129, –92/–129, –129/–1 and 4 × 20 bp sequences of *OsNAR2.1* promoter in sense orientation into the HindIII and XhoI site of min in 35Smin-LUC vector^27^. We replaced the 35S promoter sequence in the pCB302-35S-GUS vector^27^ with the –311/–129::min, –284/–129::min, –192/–129::min, –129/–1::min and the 4 × 20 bp::min, respectively. Primers are showed in Table S3.

Reporter constructs with mutated Os*NAR2.1* promoters were generated by PCR using 192::GUS plasmid of *OsNAR2.1*. For the M1, M2, M3 and M4 mutation, we got the 6 bp, 8 bp, 17 bp and 19 bp nucleotides mutation on the basis of 20 bp sequence using primers in Table S4. The cloned fragments containing the intended mutations were recovered from the resultant plasmid, and used to replace the 35S-promoter of the pCB302-35S-GUS vector.

### Rice Transformation

The constructs were obtained and transformed into callus initiated from the seeds of rice (Nipponbare) by Agrobacterium tumefaciens (strain EHA105)-mediated transformation^58^. Rice embryonic calli were induced on N_6_ media and transformation was performed by Agrobacterium-mediated co-cultivation^58^. Transgenic plants were selected on a medium containing 50 mg/L glyphosate (Roche, Indianapolis, IN, USA).

### Plant material growth conditions

Both WT and the transgenic seeds of rice (cv. *Nipponbare*) were surface-sterilized with 10% (v/v) H_2_O_2_ for 30 min and rinsed thoroughly with deionized water. The sterilized seeds were germinated on a plastic support netting (mesh 1 mm2) mounted

in plastic containers for one week. Uniform seedlings were selected and then transferred to a tank containing 8 L of IRRI nutrient solution for 4 weeks at pH 5.5. After N starved for 4 days, seedlings were grown for seven days in the culture solution for nitrate or ammonium treatment with solution refreshed every 2 days. Seedlings were then collected for the analysis of the nitrate induction of GUS activity assay. IRRI nutrient solution (1.25 mM NH_4_NO_3_, 0.3 mM KH_2_PO_4_, 0.35 mM K_2_SO_4_, 1 mM CaCl_2_·2H_2_O, 1 mM MgSO_4_·7H_2_O, 0.5 mM Na_2_SiO_3_, 20 μM NaFeEDTA, 20 μM H_3_BO_3_, 9 μMMnCl_2_·4H_2_O, 0.32 μM CuSO4·5H_2_O, 0.77 μM ZnSO4·7H_2_O and 0.39 μM Na_2_MoO_4_·2H_2_O, pH 5.5) were supplied as described previously, and replaced every two days. To inhibit nitrification, 7 μM dicyandiamide (DCD-C_2_H_4_N_4_) was mixed into all the solutions. Plants were grown in a growth chamber (Thermoline Scientific Equipment Pty. Ltd., Smithfield, Australia) at 30 °C during the day and 22 °C during the night with a 16-h light/8-h dark regime. The relative humidity was controlled at approximately 70%.

### qRT-PCR analysis of *OsNAR2.1* expression

Total RNA was isolated from the roots of rice seedlings. RNA extraction, reverse transcription, and quantitative reverse-transcription PCR (qRT-PCR) were performed as described previously^59^. Primer sets for *OsNAR2.1* (AP004023.2) as following, Forword: 5′-CAGTCGGTTTGGTTTGTCAG-3′; Reverse: 5′- TGAGGGAGGCGTGGATGC -3′.

### Quantitative measurement of GUS activity

Histochemical GUS staining was performed as described previously^27^, and quantification of the extractable GUS enzymatic activity using fluorescent substrate was carried out according to the method described by Jefferson *et al.*^60^. Samples (1–10 mg of root tissues) frozen in liquid N were disrupted for 1 min, then suspended into 1 mL GUS extraction buffer (50 mM Na_3_PO_4_, pH 7.4, 10 mM EDTA, 0.1% Triton X-100 (Sigma, St-Louis, MD, USA), 0.1% sodium lauryl sarcosine, 10 mM b-mercaptethanol). Reactions were initiated by mixing 50 mL of protein extract with 120 mL of 1 mM p-nitrophenyl-b-Dglucuronide (Sigma-Aldrich) at 37 °C for 1 to 4 h (GUS activity stayed linear for up to 16 h), and were stopped by adding 800 mL 125 mM Na_2_CO_3_, then measured with a Wallac Victor 2 spectrofluorimeter (Perkin Elmer, Waltham, MA, USA) at 355 nm excitation and 460 nm emission. Protein concentration was quantified using the Protein Assay reagent (Bio-Rad Laboratories, http://www.bio-rad.com).

## Additional Information

**How to cite this article**: Liu, X. *et al.* Two short sequences in *OsNAR2.1* promoter are necessary for fully activating the nitrate induced gene expression in rice roots. *Sci. Rep.* **5**, 11950; doi: 10.1038/srep11950 (2015).

## Supplementary Material

Supplementary Information
