# Supplementary material for: Two short sequences in OsNAR2.1 promoter are necessary for fully activating the nitrate induced gene expression in rice roots
Source: Sci Rep. 2015 Jul 7;5:11950. doi: 10.1038/srep11950 (PMC4493634; doi:10.1038/srep11950)
Supplement: Supplementary Information [file srep11950-s1.pdf]

Two short sequences in *OsNAR2.1* promoter are necessary for fully activating nitrate induced expression in rice roots

Xiaoqin Liu, Huimin Feng,Daimin Huang,Miaoquan Song,Xiaorong Fan,Guohua Xu#

Supplementary Information

Figure S1. GUS expression patterns in the transgenic seedlings which were cultured in IRRI culture solution for 4 weeks.

-311::GUS, -284::GUS, -192::GUS, -129::GUS, -311/-129::min::GUS, -284/-129::min::GUS, -192/-129::min::GUS, -129::min::GUS, M1::GUS, M2::GUS, M3::GUS, M4::GUS and 4 x 20bp::min::GUS represent the transgenic lines with different fusions of the promoter regions with GUS reporter gene and/or 35S minimum promoter. Bars, 1 mm.

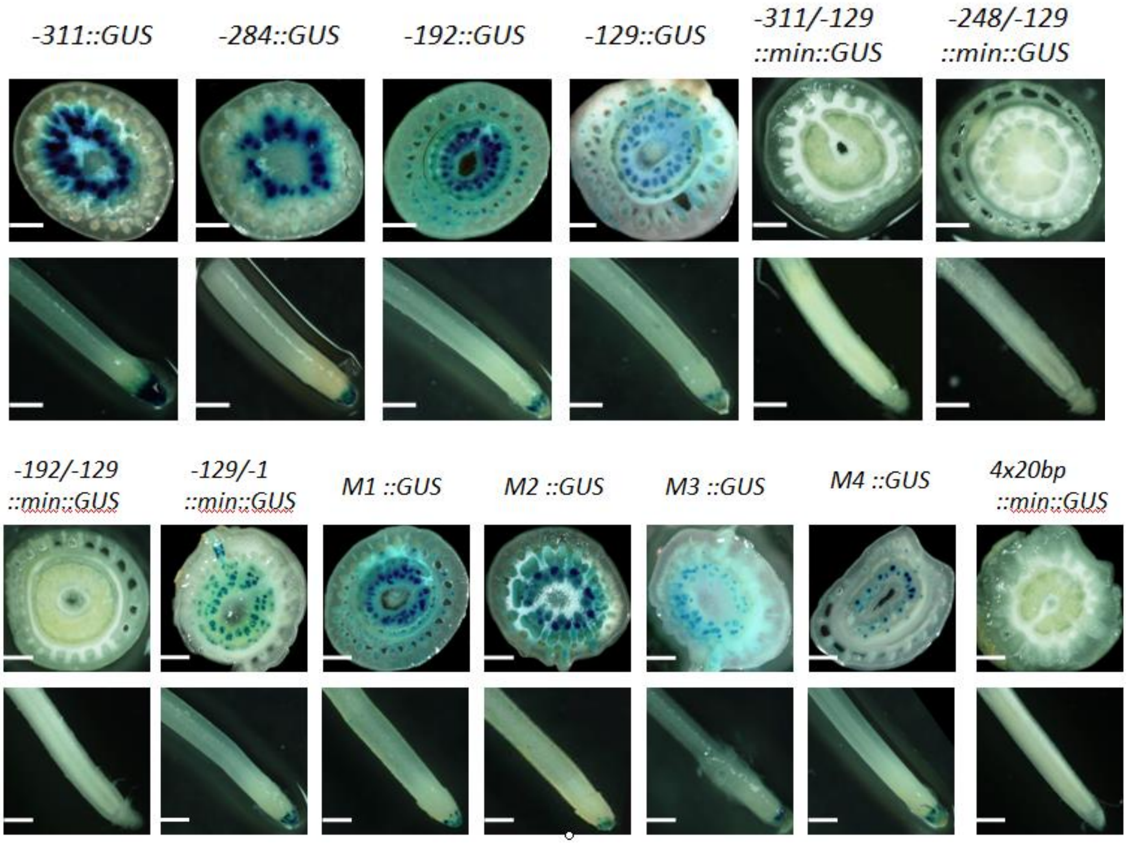

Figure S2. Relative expression of *OsNAR2.1* detected by qRT-PCR analysis in the roots.

The roots of WT and the transgenic lines by different fusions of the promoter regions with GUS reporter gene were treated with 0.2 mM NO_3^- or 0.2 mM NH_4^+. Total RNA was isolated from roots. WT, wild type. The length and mutation of the promoter for the transgenic lines are the same as presented in Figure S1 and materials and methods. Analysis was performed on five independent transgenic lines grown in either ammonium or nitrate solution. Values are mean ± SE of six biological replicates.

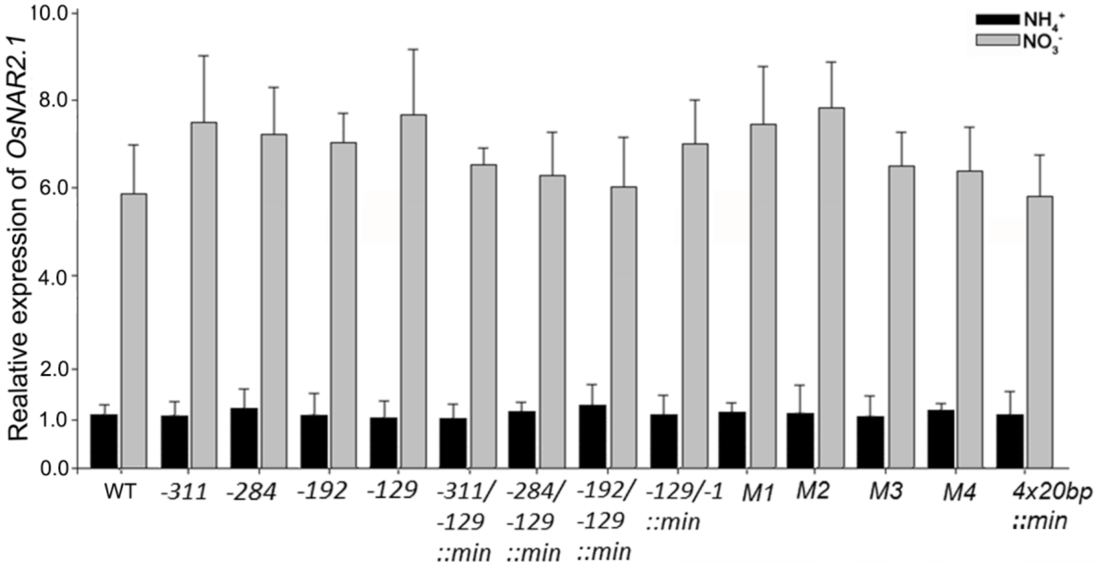

Table S1. The putative cis-regulatory elements in the -129/-1bp region of *OsNAR2.1* promoter.

**Factor identity**	**Signal Sequence**	**motif frequency***	**motif position**	**putative function**
GATABOX	GATA	3	96 (+), 98(+), 98 (-)	nitrate and light regulated, and tissue specific expression; conserved in the promoter of all LHCII type I Cab genes
MYBPLANT	MACCWAMC	3	114(-), 117(-), 98(-)	Plant MYB binding site
ANAERO2CONSENSUS	AGC	*2*	41(+), 44 (+)	anaerobic metabolism
SURECOREATSULTR11	GAGAC	*2*	73 (-), 79 (-)	sulfur-responsive element (SURE)
WRKY71OS	TGAC	*1*	123 (-)	A core of TGAC-containing W-box: WRKY superfamily of plant transcription factors
TATABOX2	TATAAAT	*1*	1 (+)	accurate transcription initiation
ARR1AT	NGATT	*1*	53 (+)	ARR1-binding element: cytokinin regulated transcription factor
INRNTPSADB	YTCANTYY	*1*	56 (+)	light-responsive transcription
PALBOXAPC	CCGTCC	*1*	83 (+)	ammonia-lyase
TATCCAOSAMY	TATCCA	*1*	98 (+)	mediation of sugar and hormone regulation
DRECRTCOREAT	RCCGAC	*1*	110 (-)	drought, high-light,cold

***occurrence in either orientation**

Table S2. The primers for construction of -311::GUS, -284::GUS, -192::GUS, -129::GUS fusions, respectively.

**Construct**	**Primer**	**Sequence (5'–3')**
-311::GUS	Forward	ca AAGCTTCCCCCAAAACCGCATGATCT
-311::GUS	Reverse	gaCCATGGTGCTGACAAACCAAACCGACT
-284::GUS	Forward	gcAAGCTTTCTCTTCTCTTCTCACACG
-284::GUS	Reverse	gaCCATGGTGCTGACAAACCAAACCGAC
-192::GUS	Forward	gcAAGCTTTGCCTCTTGAATCCAACGAA
-192::GUS	Reverse	gaCCATGGTGCTGACAAACCAAACCGAC
-129::GUS	Forward	gggcccAAGCTTTATAAATTCACCGATCGATC
-129::GUS	Reverse	gaCCATGGTGCTGACAAACCAAACCGAC

Table S3. The primers for construction of -311/-129::min::GUS, -284/-129::min::GUS, -192/-129::min::GUS, -129::min::GUS fusions, respectively.

Construct	Primer	Sequence (5'–3')
-311/-129::min::GUS	Forward	ca AAGCTTCCCCCAAAACCGCATGATCT
-311/-129::min::GUS	Reverse	gaCTCGAGGGCGGGAGAGCGAGGCTACG
-284/-129::min::GUS	Forward	gcAAGCTTTCTCTTCTCTTCTCACACG
-284/-129::min::GUS	Reverse	gaCTCGAGGGCGGGAGAGCGAGGCTACG
-192 /-129::min::GUS	Forward	gcAAGCTTTGCCTCTTGAATCCAACGAA
-192/-129::min::GUS	Reverse	gaCTCGAGGGCGGGAGAGCGAGGCTACG
-129::min::GUS	Forward	gggcccAAGCTTTATAAATTCACCGATCGATC
-129::min::GUS	Reverse	gaCTCGAGTGCTGACAAACCAAACCGAC

Table S4. The primers for constructing the fusion of 20bp sequence mutation with GUS. M1::GUS, M2::GUS, M3::GUS and M4::GUS represent different pairs of nucleotides in the 20bp sequence.

Construct	Primer	Sequence (5'–3')
M1	Forward	TCGCCGGAGCACGCCATGGAGGCTAAGCCGGTGGC
M1	Reverse	GAGCTGCACGCTGCCCACCCCGGCCGGCGACGCG
M2	Forward	GGGCCCGGGATCCTGATGGACTCGTCGACGGTGGG
M2	Reverse	GAGCTGCACGCTGCCGGCGTGCTCCGGCGAGTTGTTG
M3	Forward	CACAACATCGAGGACATGGCGAGGCTAGCCGGCG
M3	Reverse	AGATCTGAGTCCGGACTACTTGTCCTTCTTGCGC
M4	Forward	GGCAGCGTGCAGCTCGCCGAC
M4	Reverse	CAGGATCCCGGGCCCGCGGT
